# Mastoid Obliteration With Freeze-Dried Bone Allograft in Canal Wall Down Tympanoplasty: Description of a Novel Technique and Case Report

**DOI:** 10.1155/2024/4874411

**Published:** 2024-10-30

**Authors:** Antonio Faita, Giulia Montagner, Diletta Trojan, Valerio Maria Di Pasquale Fiasca

**Affiliations:** ^1^Section of Otorhinolaryngology, Cittadella Hospital, Cittadella, Italy; ^2^Fondazione Banca dei Tessuti del Veneto, Treviso, Italy; ^3^Section of Otorhinolaryngology, Department of Neurosciences, University of Padova, Padova, Italy

**Keywords:** canal wall down mastoidectomy, cholesteatoma, freeze-dried bone, homologous bone, mastoid obliteration

## Abstract

Mastoid obliteration can be performed after canal wall down (CWD) mastoidectomy with various materials. Homologous bone tissue harvested from cadaver donor represents a feasible option with advantages. The purpose of the study is to describe the case of a patient diagnosed with middle ear cholesteatoma treated with mastoidectomy of the CWD and mastoid obliteration with homologous freeze-dried corticocancellous bone particulate in the Cittadella Hospital Ear, Nose, Throat (ENT) unit. The preoperative characteristics of the patients, the procurement and processing of bone allografts, the surgical technique, and postsurgical outcomes are described. No perioperative and postoperative complications were observed, and no rejection or foreign body reactions occurred. The patient then underwent a seriated follow-up. Audiometric tests showed an improvement in hearing levels. The volume of the neoexternal ear canal was 2.01 cm^3^. The case demonstrated clinical stability, substantial hearing recovery, and no need for specialist cleaning of the reformed external ear canal (EEC). The freeze-dried bone tissue allograft, in the technical way we used, appears to be a viable option in mastoid obliteration because homologous bone is not affected by material shortage, has fast assimilation, and ensures a useful radiological examination scan, at a low cost.

## 1. Introduction

Treatment of chronic otitis media with cholesteatoma (COMC) has not reached a single gold standard. Different possible surgical procedures are available. The best choice depends on various factors, such as the position and extension of the cholesteatoma and the residual hearing functions [1].

Mastoid obliteration is a reconstructive procedure that follows a canal wall down (CWD) mastoidectomy [2]. The CWD includes drilling the posterior wall of the external ear canal (EEC), creating a common cavity between the EEC and the mastoid. Compared to other common surgical strategies, this type of procedure provides a better visualization of the tympanic cavity. CWD is most frequently indicated in the treatment of COMC [3]. Mastoid obliteration includes restoration of a posterior wall of the EEC with a functional result similar to other tympanoplasty techniques. It was described for the first time in 1911 by Mosher, using a superiorly based postauricular flap [4], whereas Palva was the first who described the use of bony material in combination with a musculoperiosteal flap [5].

Today, several surgical options are available to perform mastoid cavity obliteration, with no general consensus on standardization of procedures [6]. The use of organic materials such as free grafts, bone patè, fat grafts, cartilage, fascia [7–9], and other synthetic materials such as ceramic materials and hydroxyapatite is well established [10–12]. More recently, bioactive glass has been proposed as a feasible material for obliteration [13, 14]. Homologous bone tissue is a feasible option for reconstructive surgical procedures. Human tissue has been successfully used for reconstructive procedures in the district of the head and neck [15–19]. The bone tissue graft is harvested from donor cadaver, avoiding surgical damage to the donor site. Furthermore, it represents a feasible option in the reconstruction of wide defects, or in pediatric patients, who do not have developed the amount of tissue bone necessary for surgical procedures [20]. The safety and efficacy of freeze-dried homologous bone grafting are reported in several surgical fields, such as dentistry [21–23], maxillofacial surgery [24], and orthopedics [25].

There are no available studies describing the technical use of a freeze-dried bone tissue allograft (freeze-dried corticocancellous particulate) in obliterating the surgical cavity of mastoidectomy. We present the case of a pediatric patient diagnosed with middle ear cholesteatoma treated with mastoidectomy with CWD in the Cittadella Hospital Ear, Nose, Throat (ENT) unit. After this procedure, a mastoid obliteration was performed with homologous freeze-dried corticocancellous bone particulate.

## 2. Case Presentation

In this study we report the case of a 10-year-old male patient, with the diagnosis of recurrence of COMC of the left ear, who received secondary surgery. Two years earlier, he was treated with a CWD tympan mastoidectomy without mastoid obliteration for COMC. The disease started from an epithelial pocket in the pars flaccida of the eardrum. Preoperative hearing levels are reported in Table 1.

In COMC, epithelial debrides are trapped inside the ear. These debrides can expand over time and erode adjacent structures, causing symptoms such as hearing loss, vestibular dysfunction, facial paralysis, and other infective or abscessual complications. The presence of cholesteatoma is considered an indication for surgery.

**Figure 1:**
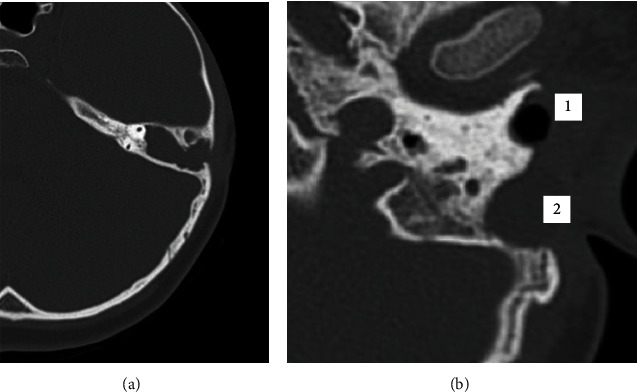
Preoperative computed tomography imaging. (A) Small posterior bone erosions compatible with resorption due to cholesteatoma. (B) Point 1, lower edge of the external auditory canal; point 2, hypointense circular signal in the mastoid compatible with cholesteatoma.

**Figure 2:**
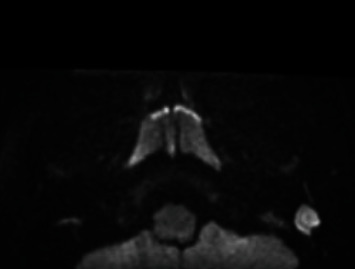
Magnetic resonance DWI sequences showing recurrence of cholesteatoma in the left ear. DWI, diffusion weighted imaging.

In the reported case, the patient had a recurrence of cholesteatoma in the entire mastoid neocavity and in the middle ear, probably caused by weakness of the reconstructed tympanic membrane and the absence of obliteration. Recurrence appeared due to clinical symptoms (otorrhea and ear pain) and partial hearing loss. Preoperative imaging with computerized tomography (CT) and magnetic resonance (MR) of the ears and temporal bones were performed. Axial CT and MR projections are shown in Figures 1 and 2.

Under general anesthesia, a retroauricular approach was performed and a muscle periosteal flap (Palva flap) was shaped. The area of the mastoid bone was extensively exposed, with a surgical inspection of the previous mastoidectomy. The anatomical boundaries (tegmen tympani, sigmoid sinus, and facial nerve) were preserved, while the cholesteatoma was removed. The bone of the posterior part of the EEC was correctly drilled in the previous CWD. The facial nerve canal was the landmark for the reconstruction of the middle ear and the obliteration of the mastoid.

The middle ear was managed with the removal of cholesteatoma in the epitympanum and phlogistic mucosa in the entire cavity, verifying the integrity of the residual ossicular chain. In CWD, the ossicular chain can be removed partially or totally; in this case, normal mobile stapes could be preserved and used to restore normal hearing. The middle ear was reconstructed using tragal cartilage juxtaposed on the head of the stapes (miringo-ossiculoplasty), posteriorly on the bone of the facial nerve canal, creating a functional smaller middle ear connected to the eustachian tube, as a classic CWD technique.

A skin plasty of the external third of the EEC was performed using two triangular local flaps sutured medially and posteriorly with the previous applied Palva flap. A large Palva flap allowed to shape subsequently two different flaps (inferiorly hinged inferior and anteriorly hinged superior local flap).

A freeze-dried bone tissue allograft (freeze-dried corticocancellous particulate) was used in the obliteration of the surgical cavity of mastoidectomy.

Human bone tissue was provided by Fondazione Banca dei Tessuti del Veneto, a nonprofit tissue bank accredited by the National Transplant Centre and Regional Competent Authority. After obtaining the proper informed consent, bone tissue was retrieved from a cadaver donor selected and screened according to Italian requirement that includes serological and molecular tests. The entire process of the human bone was conducted in the good manufacturing practice (GMP)-compliant facility of the tissue bank. The freeze-dried corticocancellous bone particulate was obtained from distal femur and proximal tibia. After retrieval, distal femur and proximal tibia were transferred in antibiotic solution, validated for human tissues decontamination [26, 27]. At the end of the first decontamination, the bones were processed removing soft tissue residues and subsequently decontaminated again. Bone tissue was ground with Fortios Bone Mill (Spierings Orthopaedics, The Netherlands); afterwards, lipids and blood residues were removed with ethanol and hydrogen peroxide (Carlo Erba, Italy). The morcellized bone was then introduced in the freeze dryer (Scientific Products, USA), and the process lasted about 18 h. Several microbiological tests were conducted throughout the process to verify the compliance with the acceptance criteria and regulation. Samples were inoculated and incubated in BD BACTEC culture vials, in accordance with the manufacturer's instructions (BD, Becton, Dickinson and Company, USA). Environmental monitoring during process was conducted according to national directives. Following the document “Guide to the quality and safety of tissues and cells for human application” (European Directorate for the Quality of Medicines and Healthcare), residual moisture content was evaluated and resulted within the recommended range of 1%–6%. The freeze-dried corticocancellous particulate was packaged and stored at +15°/+25°C until use. The retrieval process and distribution procedures were authorized by the Italian Competent Authority.

**Figure 3:**
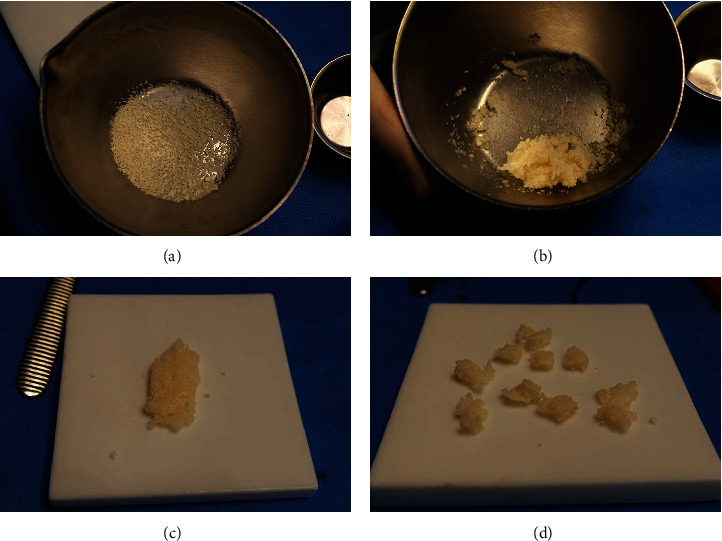
Steps for the preparation of the obliterative material: (A) freeze-dried corticocancellous particulate; (B) particulate is mixed with the human fibrin glue; (C) particulate and glue form a single block of material; and (D) the single block is divided into smaller pieces, which will be used for the obliteration of the mastoidectomy cavity.

**Figure 4:**
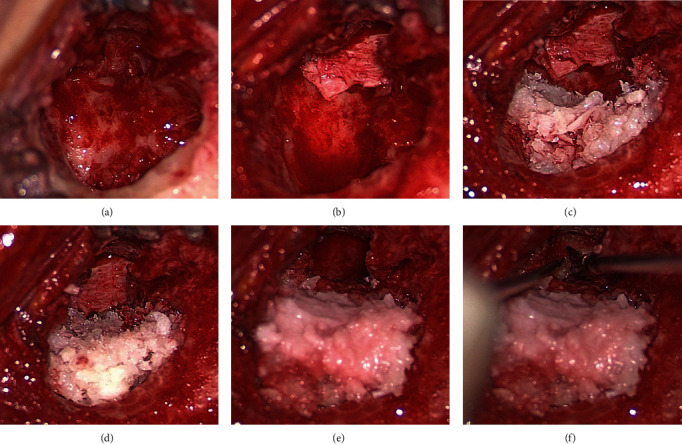
Surgical steps of mastoid obliteration: (A) mastoidectomy cavity after complete drilling of the mastoid for canal wall down (CWD) mastoidectomy; (B) first reconstructive step, positioning material for myringoplasty; (C) partial mastoid obliteration; (D) complete obliteration of the mastoid cavity; (E) the obliteration is shaped to reduce the volume of the neocavity; and (F) positioning of Spongostan to sustain the myringoplasty.

In a suitable sterile bowl, the freeze-dried corticocancellous particulate (3 cc with fine granulation) was mixed with 2 mL of human fibrin glue (Tisseel, Baxter, Italy). The still malleable compound was placed on a rigid surface, forming a tile with a height of approximately 3–4 mm; with a thin scalpel, the tile was split into approximately 5 × 5 mm blocks (Figure 3). The blocks were introduced separately into the cavity, starting medially from the depth level of the facial nerve, surrounding the superior part of the second nerve tract, the posterior and superior part of the second genu, and the posterior part of the third tract. From medially to laterally, the entire epitympanum and mastoid were obliterated with the blocks (Figure 4).

The obliteration was implemented anteriorly until a normal size EEC was newly created. The anterior level of obliteration corresponded to the new posterior wall of the EEC.

The Palva flap was flipped forward and anterior to the obliteration to promote the reepithelialization of the EEC and protect the bone obliteration from external medications and possible reabsorption. The Palva flap was long enough to be in contact with cartilage myringoplasty. When the Palva flap is smaller, other flaps can be used, such as the temporalis fascia free flap or the bovine pericardium patch, stabilized in the position between myringoplasy and Palva.

Healing of EEC and myringoplasty was supported for the following 15 days with a sterile gelatin sponge medication (Spongostan, Ethicon, USA) in the deep medial part and a laterally impregnated iodoformic gauze with chlortetracycline ointment.

After surgery, the patients underwent seriated postoperative follow-up evaluations at 15, 30, and 90 days. During this period, repeated audiometric tests and impedancemetries were performed. The postoperative EEC was calculated. The normal EEC volume has a range between 0.6 and 2.05 cm^3^

After the surgical procedure, the patient remained in our clinic and was discharged 48 h after the operation. No perioperative and early postoperative complications were observed.

The patient then underwent a seriated follow-up during the early postoperative period. The first postoperative evaluation and medication occurred in 15 days, with removal of iodoformic gauze. The second medication occurred in 22 days, after domiciliary instillation of topical ciprofloxacin. No otorrhea or sovrainfection of the site was observed.

The third check control occurred within 30 days, resulting in a complete re-epithelization of EEC, and no other medications were necessary. The first postoperative audiometry was performed in 30 days and reported a pure tone average (PTA) of 31.25 dB (Table 2).

A second postoperative audiometry was performed in 90 days: PTA was 20 dB (Table 2). The postoperative volume of the EEC was measured in the 1-month evaluation: left ear 2.01 cm^3^ and right ear 1.63 cm^3^.

**Figure 5:**
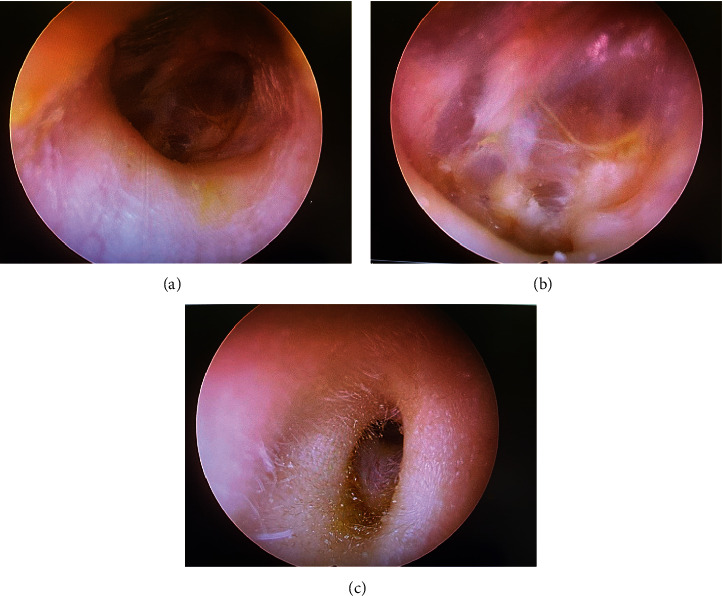
Postoperative evaluation at 1 year after surgery: (A) fully re-epithelized posterior wall of the external ear canal; (B) reconstructed tympanic membrane; and (C) external aspect of the external ear canal.

The follow-up of cholesteatoma needed a clinical examination 1 year after the surgical act, confirming the volume of the EEC volume and the PTA. No residual or recurrence of cholesteatoma was observed. However, the ear self-cleansed as the nonoperated side, and no accumulation of cerumen was observed (Figure 5).

## 3. Discussion

COMC is the most clinically aggressive type of chronic otitis media. It has the ability to be erosive for nearby structures, including the compact bone of the otic capsule. It is cause of recurrent infections and can lead to hearing loss, vestibular dysfunction, facial paralysis, and intracranial invasion [28]. The origins of cholesteatoma can be divided into congenital and acquired. The first is typically found in children and depends on embryological entrapment of squamous tissue inside the middle ear, which is present at birth [29]. Acquired cholesteatoma can be found in both adults and children. It is generated most frequently by the absorption of keratinized tissue in the middle ear through a pocket of the tympanic membrane and then a perforation. This process is caused most frequently by dysventilation of the middle ear due to eustachian tube dysfunction [30].

The treatment of COMC is surgical. The aim of surgery is the complete eradication of the disease and the prevention of recurrences [1]; secondary objective is to shape a stable, self-cleaning ear and restore good hearing.

Various surgical options are available. The most frequently applied classification of middle ear surgery depends on the management of the posterior bony wall of the external auditory canal. The procedures are defined as “canal wall up” if the posterior wall remains intact and CWD if the wall is drilled. These types of surgery have different characteristics. The canal wall up mastoidectomy provides fewer healing problems, with no cleaning issues and less need for a seriated follow-up evaluation [31]. In the CWD procedures, a more extended drilling is performed. This ensures better exposure to the structures of the middle ear, with high rates of eradication and fewer risk of residual cholesteatoma or recurrence (4%–17%) [1].

On the other hand, a large neocavity is characterized by the need for frequent specialized evaluation for clearance, a more relevant risk of chronic or recurrent otorrhea and infections, and a general worse auditory outcome.

The mastoid obliteration procedure after the performance of a CWD mastoidectomy allows for the shaping of a smaller neocavity, with great advantages [32]. It reduces the risk of persistent otorrhea and infections [33], the accumulation of cerumen in the neocavity and therefore the frequency of follow-up evaluation for ear cavity cleaning [34], and the ear sensitivity to vertigo by caloric stimulus [35]. It has a favorable impact on the rate of recurrence of cholesteatoma, compared to the results of canal wall up mastoidectomy [36–38].

The case we present demonstrated, over the years, clinical stability, substantial hearing recovery, and no need for specialist cleaning of the restored EEC. Furthermore, mastoid obliteration can be performed after both primary and secondary revision surgery [34, 39] and in first or second look surgeries. It should be considered a feasible procedure even in pediatric patients, as our case [40].

CWD tympanomastoidectomy with mastoid obliteration showed outcomes as canal wall up procedures in terms of quality of life [36]. Compared to nonobliterated CWD cavities, patients who underwent mastoid obliteration appear to show better hearing results, with similar [39, 41] or lower average air bone gap (ABG) in PTA after surgery [42]. It also provides a better hearing aid and reduces postoperative complaints such as pain, dizziness [42], and discharge [39]. The smaller cavity also allows for a faster and better healing process, as shown by epithelization of the neocavity [42].

This is the first report on the use of freeze-dried corticocancellous bone granules in mastoid obliteration. The advantages of the use of bone allografts are the greater availability of material and the elimination of donor site morbidity with respect to autogenous bone. Osteoconductive properties, absence of immunological response, or signs of inflammation are also reported after freeze-dried homologous bone particulate grafting [43, 44]. We also observed an absence of reabsorption over time.

We associated bone mastoid obliteration with the use of cartilage for tympanic membrane reconstruction, to improve ossiculoplasty and hearing outcome. Furthermore, the use of Tutopatch and Palva flaps stimulated healing and rapid re-epithelization of the EEC, preventing the exposure of the bone allograft to the outside.

Compared to bone patè, an autologous material (normally harvested from bone dust made by drilling the cortical temporal bone), homologous bone is not affected by material shortage. Assimilation to the original tissue is usually fast, less than 2 months in this first experience. The use of analogous material for reconstruction of the drilled mastoid bone ensures a useful radiological examination scan, compared to the use of other types of organic tissue. The bone preparation process ensures a complete reduction of the risk of rejection or foreign body reaction, which were not detected in the present patient up to the latest follow-up.

Many other materials have been proposed to fill the neocavity shaped by the CWD mastoidectomy (Table 3). These materials have different characteristics: biological grafts and flaps typically have good biocompatibility and low costs. On the contrary, they can undergo long-term modifications, such as reabsorption, shortening, fibrosis, and necrosis, with the possible need for revision surgeries. In those cases, they are picked up by patients (autologous), such as cartilage, muscle, and compound flaps, fat, and bone patè as cited, and cause morbidity at the donor site with more surgical procedures and risks. Moreover, they can be difficult to harvest in the correct amount, especially in revision surgeries or pediatric patients[11, 45]. Some recent studies have proposed the use of bone patè mixed with platelet-enriched plasma. This kind of mixed material has been applied to provide better wound healing and tissue sealing, with favorable results at low-costs [46].

Heterologous bone (hydroxyapatite) has been widely applied for mastoid obliteration, and it is considered to have the best osteoconductivity among nonautologous materials. The hydroxyapatite extrusion rate is high: 15.8%. Synthetic materials are usually biocompatible and can guide the process of reossification. Possible burdens associated with such materials are middle ear or bone infections and graft extrusion in middle or long-term follow-up [47]. Extrusion is a complication that affects a small percentage of synthetic materials: 0.5–3.3 Bonealive glass and 3.5%–5% silicone [11]. Different materials have different characteristics. Silicone has been applied in the shape of small blocks for reconstruction after CWD mastoidectomy. It is a low-cost material but can cause a foreign body reaction in predisposed patients [48]. Bonealive glass is a material whose study has recently spread. It is a solution composed of glass granules that are capable of chemically bonding to the surrounding bone and promoting new bone formation in the reconstruction area. A study by Fassone et al. estimated a re-epithelization time of 45 days, with low complication rates [14].

A recent study demonstrates poor results in using homologous bone, with resorption of the material leading to a 40.0% revision surgery rate. [49] However, the technical method of applying the material is not described, so it is not possible a real comparison to our technique.

In our center experience, freeze-dried bone tissue allograft appears to be a viable option in mastoid obliteration. The use of bone, mixed with the fibrin glue, can be safely associated with heterologous material and local flaps (Palva) to stimulate EEC re-epithelization. As presented in the paper, the technique and materials show some advantages: it ensures a correct assimilation into the original tissue, with clear radiological imaging and low risk of extrusion. Its availability is not affected by the dimension of the surgical site, making it a good option for patients with a narrow cortical bone, such as children. CWD with mastoid obliteration is a good surgical option for treating COMC obtaining a stable, functional, and safe ear.

## Figures and Tables

**Table 1:** Preoperative hearing levels before the CWD procedure (performed in 2019).

Hearing level	Side	Results
Preoperative air conduction PTA	Left	31.25 dB
Preoperative bone conduction PTA	Left	5 dB
Air–bone gap	Left	26.25 dB
Air conduction PTA	Right	11.25
Bone conduction PTA	Right	11.25

Abbreviations: CWD, canal wall down; PTA, pure tone average.

**Table 2:** Postoperative hearing levels at 30 and 90 days after surgery.

Hearing level	Side	30-day follow-up (dB)	90-day follow-up (dB)
Postoperative air conduction PTA	Left	31.25	20
Postoperative bone conduction PTA	Left	12.50	8.75
Air–bone gap PTA	Left	18.75	8.75
Air conduction PTA	Right	10	10
Bone conduction PTA	Right	5	5

Abbreviation: PTA, pure tone average.

**Table 3:** Main characteristics of different materials used for mastoid obliteration.

Material	Type	Advantages	Disadvantages
Homologous bone (freeze-dried corticocancellous bone)	Biological	High biocompatibility, low costs, high osteoconductivity	Additional costs compared to autologous bone
Autologous bone (bone patè)	Biological	High biocompatibility, low costs, high osteoconductivity	Possible morbidity of the donor site, shortage of material
Cartilage	Biological	High biocompatibility, low costs	Possible morbidity of the donor site, shortage of material
Fat	Biological	High biocompatibility, low costs	Possible morbidity of the donor site, reabsorption
Heterologous bone (hydroxyapatite)	Biological	High biocompatibility, high osteoconductivity	Higher costs than homologous bone, risk of postoperative extrusion
Silicone	Synthetic	Low costs	Possible foreign body reaction
Bioactive glass	Synthetic	High biocompatibility, high osteoconductivity	High costs

## Data Availability

Data supporting the findings of this study are available on request from the corresponding author. The data are not publicly available due to privacy or ethical restrictions.
